# Research Progress of Bioinspired Structural Color in Camouflage

**DOI:** 10.3390/ma17112564

**Published:** 2024-05-27

**Authors:** Yimin Gong, Haibin Wang, Jianxin Luo, Jiwei Chen, Zhengyao Qu

**Affiliations:** 1 School of Materials Science and Engineering, Hubei Polytechnic University, Huangshi 435003, China; 225011@hbpu.edu.cn; 2 School of Materials Science and Engineering, Hunan Institute of Technology, Hengyang 421002, China; hbwang@hnu.edu.cn; 3 State Key Laboratory of Silicate Materials for Architectures, Wuhan University of Technology, Wuhan 430070, China; quzhengyao@whut.edu.cn

**Keywords:** bioinspired, structural color materials, optical mechanism, color changing, camouflage

## Abstract

Bioinspired structural color represents a burgeoning field that draws upon principles, strategies, and concepts derived from biological systems to inspire the design of novel technologies or products featuring reversible color changing mechanisms, with significant potential applications for camouflage, sensors, anticounterfeiting, etc. This mini-review focuses specifically on the research progress of bioinspired structural color in the realm of camouflage. Firstly, it discusses fundamental mechanisms of coloration in biological systems, encompassing pigmentation, structural coloration, fluorescence, and bioluminescence. Subsequently, it delineates three modulation strategies—namely, photonic crystals, film interference, and plasmonic modulation—that contribute to the development of bioinspired structural color materials or devices. Moreover, the review critically assesses the integration of bioinspired structural color materials with environmental contexts, with a particular emphasis on their application in camouflage. Finally, the paper outlines persisting challenges and suggests future development trends in the camouflage field via bioinspired structural color.

## 1. Introduction

The modern high-tech, information-dense battlefield presents a complex environment characterized by complex topography, dynamic weather conditions, optimized operational efficiency, and a wide array of investigation technologies [1,2]. With the necessity to conceal military assets and minimize casualties, the development of military camouflage and stealth technology is steadily evolving towards diversification, standardization, technological sophistication, intelligence integration, and precise alignment with specific targets [3,4]. Current camouflage technology can be delineated into three primary categories: firstly, methods aiming to render the coloration of the target object congruent with the background color; secondly, techniques endeavoring to harmonize the camouflage coloration of the target object with its surroundings; and thirdly, approaches employing floral or spot patterns that juxtapose against the background color scheme. Notwithstanding these advancements, conventional camouflage painting methodologies frequently demonstrate inadequacy when confronted with a spectrum of environmental backgrounds and varied battlefield scenarios [5,6]. Thus, there is a pressing need to innovate and develop novel camouflage technologies that minimize disparities between targets and backgrounds, particularly in optical and thermal infrared spectra, to enhance adaptability on the multifaceted and dynamic battlefield [7,8].

In pursuit of advanced camouflage technologies, researchers draw inspiration from a plethora of natural phenomena, including the remarkable color-changing abilities observed in various species [9]. It is generally known that over billions of years of evolutionary processes, a multitude of organisms in the natural world have evolved the ability to camouflage themselves through adaptive alterations in body color or morphology, facilitating perfect integration with their surroundings [10]. This evolutionary adaptation serves a myriad of biological functions, encompassing mating, reproduction, hunting, and predator evasion, collectively enhancing survival capability [11,12]. The study of nature has provided insights into the intricate mechanisms underlying biological color changes and adaptations, spurring endeavors aimed at replicating these structural characteristics for the development of adaptive and practical camouflage materials [6,13,14].

A primary challenge in developing bioinspired camouflage materials lies in achieving dynamic color changes that closely resemble those found in nature [15] or realizing transparency that matches the surrounding environment [16,17,18]. While traditional camouflage relies on static patterns or coloration, bioinspired approaches aim to create materials capable of mimicking nature or responding in real-time to changing conditions. This necessitates a deep understanding of the underlying physics and chemistry of natural coloration, along with innovative engineering techniques to replicate these processes in artificial materials [19]. Recent advancements in materials science and nanotechnology have facilitated significant progress in this area. By leveraging principles of photonic crystals, nanostructures, and responsive polymers, scientists have created artificial materials capable of dynamic color changes in response to external stimuli such as light [20], temperature [21], or humidity [22,23]. These materials can emulate the vibrant color found in nature, offering new avenues for camouflage and stealth technology. Furthermore, the integration of bioinspired camouflage materials with advanced sensor technologies holds promise for enhancing situational awareness and threat detection on the battlefield. By incorporating sensors capable of detecting changes in environmental conditions or enemy movements, adaptive camouflage systems could dynamically adjust their coloration to evade detection by both human observers and surveillance equipment.

This manuscript embarks on an exploration of natural colors, elucidating the mechanisms governing color transformations. Drawing inspiration from nature, artificial structural color materials are meticulously engineered, showcasing dynamic color transitions orchestrated by external stimuli. The investigation then delves into the technology of merging and modulating bioinspired structural colors with environmental background color. The resulting structural color camouflage materials introduce innovative perspectives, offering novel solutions to address the evolving requirements of the next generation of camouflage materials.

## 2. Structural Colors in Natural Creatures

**Figure 1:**
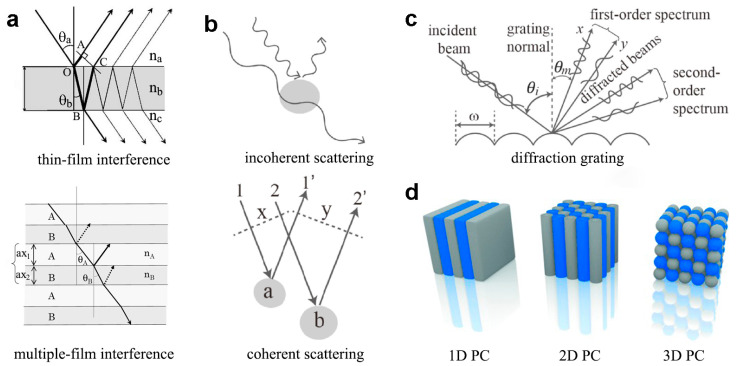
(**a**) Film interference including thin-film and multilayer interference. Reproduced with permission [32]. Copyright (2008) IOP Publishing. (**b**) Scattering including incoherent scattering and coherent scattering. (**c**) diffraction grating. (**b**,**c**) Reproduced with permission [50]. Copyright (2023) Elsevier. (**d**) photonic crystals (one-, two- and three- dimensional photonic crystals (1D, 2D, 3D PC)). Reproduced with permission [51]. Copyright (2014) Wiley-VCH.

In the realm of natural phenomena, the exhibition of vibrant colors is facilitated through four mainly distinct manifestations: pigmentation, structural coloration, fluorescence, and bioluminescence [24,25,26,27]. While certain instances may rely solely on one form for color rendering, there are occasions where a synergistic collaboration among these mechanisms is requisite to achieve optimal visual effects [19]. Pigmentary color originates from the selective absorption of light by pigment molecules, dyes, or metals [28,29,30], whereas structural colors emerge from the intricate interplay between incident light and specific micro- or nanostructured materials [11,12]. These structural colors possess distinctive characteristics, notably the exhibition of enduring color stability [31]. Presently, the manifestation of natural structural colors is governed by four principal mechanisms: film interference [32], diffraction grating [33], light scattering [34,35], and photonic crystals [36,37] (Figure 1). These mechanisms elucidate the intricate interplay of light and matter, offering profound insights into the fundamental principles underlying the generation of colors observed in nature. Such comprehension not only fuels advancements in fundamental research but also inspires innovative applications across various disciplines, from materials science to photonics, with implications for technology development and biomimetic design.

The prevailing color phenomenon within the natural domain is represented by structural colors based on film interference, which encompass both thin-film and multilayer interference. Instances of coloration resulting from thin-film interference are observable in *slime molds* [38], while the existence of multilayer interference in the tortoise beetle gives rise to structural colors characterized by brighter and more saturated compared to those originating from thin-film interference [39]. Notably, diffraction grating finds its most frequent manifestation in invertebrates, as evidenced by the iridescent chromaticity observed in butterflies [40,41]. The concept of scattering elucidates the phenomenon wherein light waves, while traversing a transparent medium, deviate from their original trajectory due to inherent microscopic non-uniformities in said medium. An illustrative example includes the manifestation of white coloration on beetles’ integuments, attributable to the scattering of light by nanoscale particles [42,43,44]. With the first discovery of two-dimensional (2D) and three-dimensional (3D) photonic crystal structures in *sea mouse* [45] and *weevil* [46], respectively. Researchers are delving deeper into the exploration of natural phenomena. Currently, photonic crystal structures have been identified in diverse marine organisms, insects, and avian species [47,48,49].

In nature, chitin, keratin, and melanin granules possess the inherent capability to assemble nanostructures, resulting in the manifestation of structural colors [52,53,54]. Such colors exhibit notable saturation and endure for extended durations, contingent upon the preservation of the ordered nanostructures. Nevertheless, these structural colors are characterized by a deficiency in self-regulatory mechanisms, thereby falling short of meeting the demands imposed by contemporary military camouflage criteria.

**Figure 2:**
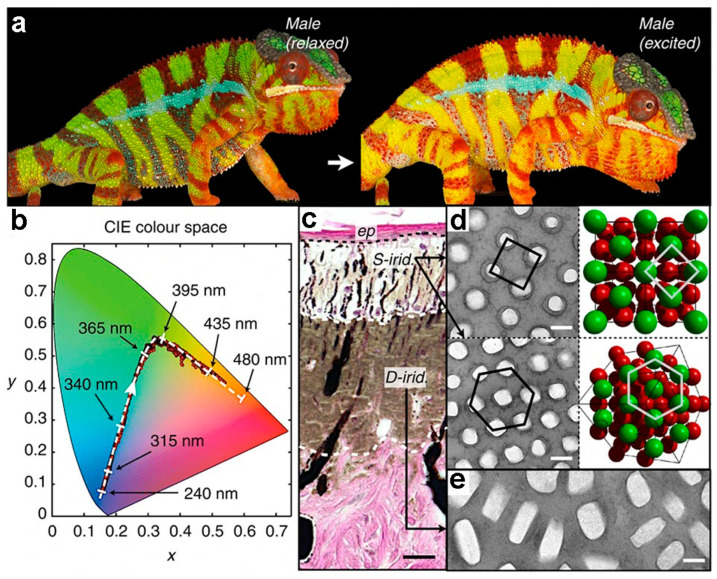
(**a**) Male chameleons exhibit reversible color changes, transitioning from green in a relaxed state to yellow when excited, with blue vertical bars beneath red pigment cells. (**b**) red dots: CIE chromaticity chart displaying the temporal evolution of color; dashed white line: numerical simulations using a face-centered cubic (FCC) lattice of guanine crystals and black arrows for lattice parameter. (**c**) Cross-section of chameleon skin, revealing the epidermis (ep) and two thick layers of iridophores. TEM images of guanine nanocrystals in (**d**) S-iridophores alongside a three-dimensional model of an FCC lattice and (**e**) D-iridophores. Scale bars, 20 mm (**c**); 200 nm (**d**,**e**) [58]. Copyright (2015) The Authors.

The dynamic modulation of structural colors in biological organisms is predominantly contingent upon alterations in surface microstructure under varying conditions to attain desired camouflage effects [55,56,57]. Species such as *chameleons* [58], *cephalopods* [59], *fish* [60], and *beetles* [61] exhibit the capacity to modify their body color and visual characteristics in response to environmental stimuli. Throughout the course of evolution, the surface microstructure not only engenders structural colors via distinct reflection within the visible light spectrum but also assumes a regulatory role in the near-infrared spectrum. Teyssier et al. [58] revealed that the color transformations observed in chameleons are a result of the synergistic effects of pigment colors and structural colors (Figure 2a,b). The skin composition of panther chameleons comprises two superposed thick layers of iridophore cells (Figure 2c), containing guanine crystals exhibiting distinct sizes, shapes, and organizations. The upper layer encompasses superficial (S-) iridophores housing small, closely packed guanine crystals (diameter 127.4 ± 17.8 nm) organized in a triangular lattice (Figure 2d). Notably, the lattice spacing of these S-iridophores expands upon stimulation, leading to a Bragg diffraction peak shift towards longer wavelengths, thereby inducing a color change from green to red in the chameleon’s skin. The second layer, comprising deep (D-) iridophores, features larger brick-shaped guanine crystals (length 200–600 nm, height 90–150 nm) with a somewhat disorganized arrangement. This layer reflects a considerable portion of sunlight, particularly in the near-infrared range. The dynamic structural coloration mediated by guanine in these iridophores holds significant relevance in interspecies communication, such as during combat and courtship activities in chameleons, and may also play a role in the thermoregulation process. Gur et al. [62] elucidated that *Sapphirina metallina* males exhibited a magenta hue in their dark-adapted state, transitioning to yellow when exposed to light. In both dark-adapted and light-adapted states of *S. metallina*, the crystal thickness remained approximately ≈70 nm, while the cytoplasm thickness varied from 190 ± 8 nm in the dark-adapted state to 135 ± 5 nm in the light-adapted state. This observation suggests that alterations in cytoplasmic spacings modulate the reflected colors. Importantly, guanine crystals are pervasive in the photonic crystal structures across a range of biological organisms [63].

Moreover, the dynamic chromatic transformations observed in cephalopods, including species such as *octopuses*, *cuttlefish*, and *squids*, involve a complex interplay between pigment colors and structural colors [59,64]. Situated atop the corium layer proximate to the cuticle, the chromatophores serve as sites for pigment molecule generation [65]. Beneath the chromatophore layer lie iridophores and leucophores responsible for producing structural color effects. The orderly arrangement of structures within iridophores constitutes a Bragg Reflector, enabling color modulation, while the presence of white granules in leucophores induces broad-spectrum diffusive reflection and scattering of light [59].

## 3. Bioinspired Materials with Tunable Structural Colors

Through a progressive exploration of the natural world, researchers have systematically revealed the complexities governing organisms’ color-adaptive abilities. Building upon this understanding, a range of bioinspired devices was developed, aiming primarily at achieving camouflage and concealment. The preparatory methodologies primarily revolve around modifying the structural characteristics of photonic crystals, film interference, and plasmonic elements, thereby inducing significant alterations in chromatic properties.

### 3.1. Photonic Crystal Modulation

The optical characteristics inherent in 1D photonic crystals adhere to the Bragg equation mλ = 2ndsin*θ* [66]. Through the assembly of photonic crystal building blocks or coloration units within responsive polymers, composite photonic crystal materials can be systematically crafted [67]. When exposed to diverse external stimuli, these stimuli induce alterations in the lattice constants (*d*) [68], effective refractive index (*n*) [69] and crystal relative orientation [70] within the composite material. Subsequently, such modifications give rise to shifts in peak positions, peak intensity, peak shape or the full-width at half-maxima (FWHM) of peak, ultimately manifesting macroscopically as alterations in coloration.

**Figure 3:**
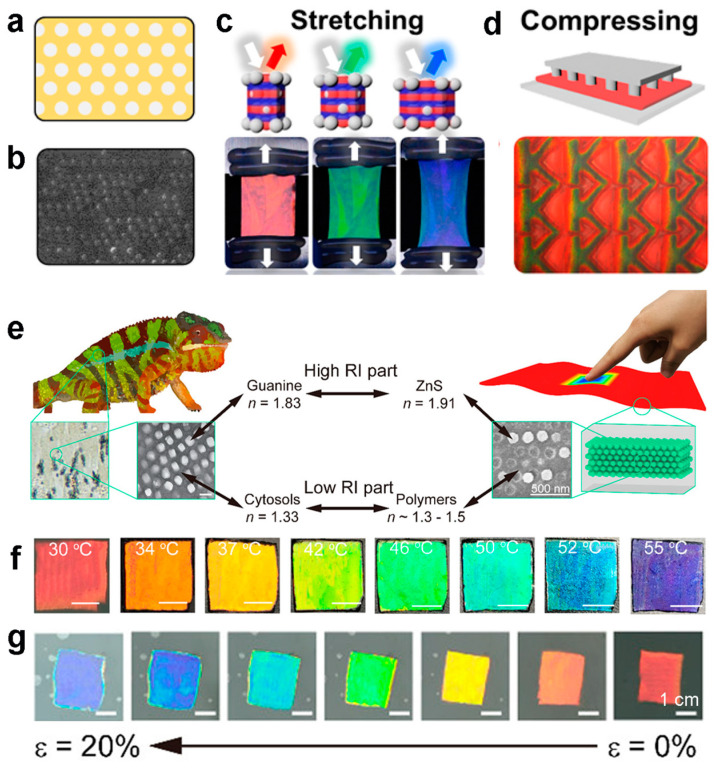
(**a**) Schematic of mechanochromic photonic films composed of a non-close-packed array of silica particles embedded in an elastomeric matrix. (**b**) SEM of the film cross-section. (**c**) Schematic of the stretching of photonic film and corresponding photographs. (**d**) Schematic of the compressing of photonic film and corresponding photographs. (**a**–**d**) Reproduced with permission [71]. Copyright (2017) American Chemical Society. (**e**) Schematic of artificial chameleon skin. Digital photographs of the thermochromic PC film under different (**f**) temperatures and (**g**) compression. (**e**–**g**) Reproduced with permission [72]. Copyright (2021) American Chemical Society.

Drawing inspiration from the guanine crystal structure arrangement observed in chameleon skin, Lee et al. devised a mechanically adaptive film featuring a non-close-packed three-dimensional photonic crystal structure [71]. The dense solvation layer facilitates the dispersion of high concentrations of silica particles in a rubber precursor of poly(ethylene glycol) phenyl ether acrylate (PEGPEA), ensuring high stability against agglomeration and spontaneous formation of a photonic structure, as illustrated in Figure 3a. Upon photopolymerization of the dispersion, the resulting composite films exhibit pronounced reflection colors. Scanning electron microscopy analysis of the film’s cross-section (Figure 3b) reveals the embedding of non-close-packed silica particles within the film. Stretching the film induces alterations in thickness, thereby prompting a shift in the lattice constant and consequent blue shift in color across the entire visible light spectrum (Figure 3c). The non-close-packed arrangement of silica particles confers the film with a stretching strain capacity of up to 70%, while minimal particle rearrangement ensures complete reversibility of color along the strain direction, highlighting the film’s elastic deformation without residual strain. Moreover, compressive strain also results in discernible color changes, as depicted in Figure 3d. Similarly, Wu et al. mimicked the high refractive index contrast observed in guanine nanocrystals and cytosols. They utilized a two-step filling technique to immobilize ZnS nanoparticles within responsive polymers, resulting in the creation of an artificial chameleon skin characterized by a non-close-packed structure (Figure 3e) [72]. Through the utilization of poly(N-isopropylacrylamide) hydrogel, alterations in hydrophobicity are elicited in response to temperature fluctuations. These changes precipitate volume expansion and contraction, subsequently engendering adjustments in lattice constant and resultant color variations (Figure 3f). Capitalizing on the benefits of this non-close-packed configuration, noticeable color changes are also observed during the compression process (Figure 3g).

**Figure 4:**
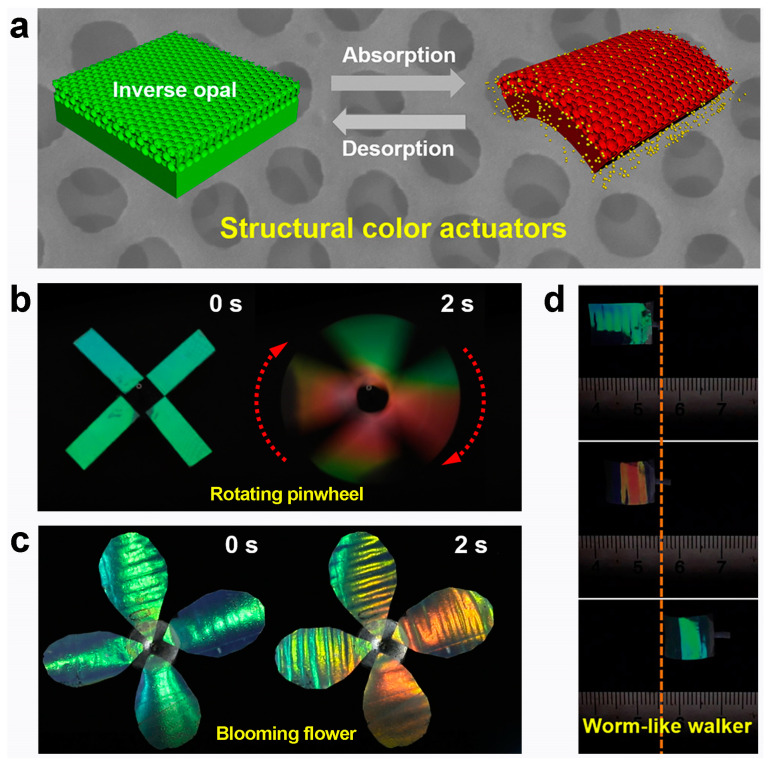
(**a**) Schematic of the structural color actuators. (**b**) The non-patterned structural colored pinwheel rotated with a bright and vivid color change under nitrogen-acetone mixed flow. (**c**) The patterned structural colored flower blooms and closes with different vivid colors under acetone flow. (**d**) Worm-like walker with accompanying dynamic color alterations. Reproduced with permission [73]. Copyright (2019) Elsevier.

Wang et al. [73] presented structural-color actuators designed to replicate the dynamic behavior of chameleons, providing vivid color alterations and programmable shape transformations in response to environmental. Fabricated using inverse opal poly(trimethylolpropane triacrylate) (PTMPTA) films, these actuators leverage highly ordered silica colloidal crystals as structural templates (Figure 4a). PTMPTA was selected for its rapid absorption and desorption properties concerning organic solvents. Exposure to a mixed flow of nitrogen and acetone induces continuous rotation of the structural colored pinwheel, accompanied by rapid transitions between bright green and orange (Figure 4b). Furthermore, an artificial flower-shaped actuator featuring patterned PHEMA stripes demonstrates the ability to open, close, and undergo dynamic multicolor changes upon exposure to or removal of acetone vapor (Figure 4c). Additionally, the authors presented a soft walker prototype capable of spontaneous propulsion when intermittently exposed to acetone flow, with corresponding color changes from green to orange-red observed in the walker (Figure 4d). While combining responsive materials with photonic crystal structures can develop structural-color actuators that mimic the behavior of chameleons, liquid crystalline polymers (LCPs) offer remarkable reversible stimuli-responsive shape-morphing properties, making them ideal candidates for preparing structural color actuators for camouflage applications [74,75,76]. Recently, several liquid crystal-based structural color actuators have been developed [77,78]. For instance, Li et al. [79] utilized self-assembled cellulose nanocrystals (CNCs) and a polyurethane (PU) substrate to develop a humidity/infrared-sensitive intelligent actuator with synergistic structural color changes. Additionally, Sol et al. [80] created a scallop-inspired actuator with 3D-programmed structural color using cholesteric liquid crystals (ChLC) oligomer ink.

**Figure 5:**
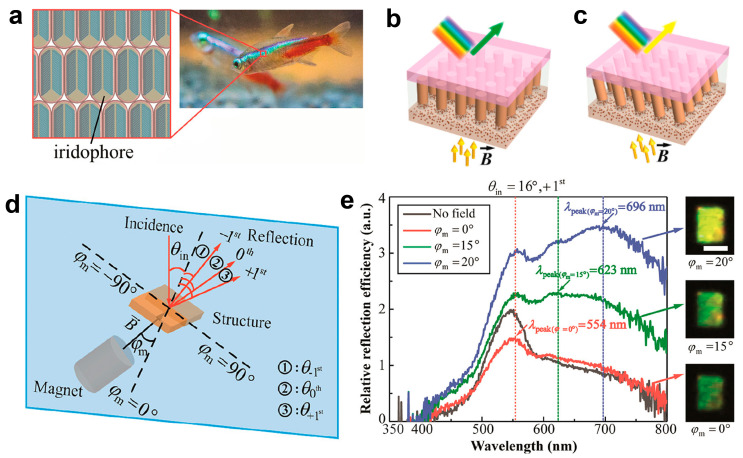
(**a**) Illustration of a neon tetra fish and its photonic structures, with iridophore arrays within the lateral stripe. Illustration of periodic self-assembled columns (SACs) under (**b**) a vertical magnetic field and (**c**) a tilted field, respectively. (**d**) Schematic illustrating reflection diffraction with various orders when an incident light beam with angle *θ*_in_ is applied. (**e**) Spectral analysis of the +1st order reflection diffraction at *θ*_in_ = 16°, showcasing a notable red-shift, validated by camera images on the right side., scale bars, 1 mm. Reproduced with permission [85]. Copyright (2019) American Chemical Society.

As mentioned above, researchers have presented the fabrication of photonic structures, necessitating external stimuli such as stretching, compressive strain, and temperature-induced volume variations to induce color changes in photonic crystal materials. This observed color modulation conforms to the “accordion” model, which posits a dependence on large strains for the color changes. But it is important to acknowledge that the “accordion” mechanism governing lattice constant adjustments may raise concerns regarding structural stability or buckling of the materials [9,81,82,83]. The color modulation exhibited by the tropical fish *neon tetra* aligns with the “venetian blind” model, wherein color manipulation relies on altering the tilt of the crystal array [84]. Drawing inspiration from this phenomenon (Figure 5a), Luo et al. devised a mechanism to achieve dynamic iridescence that can be magnetically controlled (Figure 5) [85]. This methodology entails utilizing a 2D periodic magnetic nanopillar array as a template to facilitate the assembly of iron oxide nanoparticles within a liquid environment. Under the influence of an external magnetic field, the nanopillar array generates a periodic local field, guiding the self-assembly of iron oxide nanoparticles into periodic self-assembled columns (SACs). Additionally, the local field exerts an “anchor effect”, immobilizing the base of SACs and enabling their tilting about the base to induce color changes (Figure 5b,c). The incident light at angle *θ*_in_ initiates discrete diffraction orders according to Bragg’s law, as illustrated in Figure 5d. Utilizing a white light source at *θ*_in_ = 16°, the spectrum and optical images were recorded in Figure 5e. Observations reveal a transition in color appearance of the +1st order from dark green to yellow, accompanied by a red-shift upon tilting the field from 0° to 20°. Notably, at *φ*_m_ = 15°, the peak wavelength of the +1st order measures 623 nm, yet the sample’s perceived color does not align with the red hue, potentially attributed to an additional prominent peak near 550 nm originating from the diffraction of the FFPDMS template, consequently rendering the perceived color as yellowish-green. Furthermore, at *φ*_m_ = 20°, the peak wavelength of the +1st order surpasses the visible spectrum, concomitant with the sample’s color manifestation as yellow, indicative of a secondary peak in the green spectrum. While this approach alleviates the need for substantial strains in the “accordion” mechanism to achieve comprehensive tuning of the visible light spectrum, it does present the drawback of low color saturation.

### 3.2. Plasmonic Modulation

**Figure 6:**
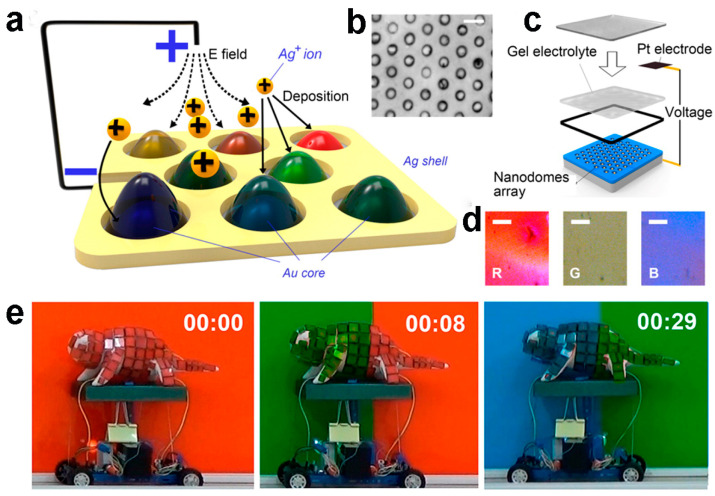
(**a**) Schematic diagram of the plasmonic cell. (**b**) Top-view SEM image of the Au nanodome array. Scale bar: 100 nm. (**c**) Schematic diagram of the working plasmonic cell. (**d**) Microscopic image of the device’s color in RGB color. Scale bar: 50 μm. (**e**) The demonstration of plasmonic chameleon. (**a**–**e**) Reproduced with permission [89]. Copyright (2016) American Chemical Society.

In subwavelength nanostructures, the phenomenon of surface plasmon resonance entails the absorption or emission of specific frequency bands within the visible light spectrum, giving rise to plasmonic structural colors [86]. In recent years, researchers have developed various techniques for the fabrication of plasmonic colors [87,88]. For instance, Wang et al. implemented a 3D-printed chameleon model enveloped with an active localized surface plasmon resonance device comprised of nanoscale gold particle arrays, emulating the distinctive armor scales of a chameleon [89]. By applying voltage to the scales, various thicknesses of silver films are selectively deposited or removed on the gold nanoparticles, thereby modulating the properties of the localized surface plasmons. This dynamic process facilitates real-time light reflection at distinct wavelengths, thereby affecting color changes (Figure 6a–d). The mechanically color-changing chameleon incorporates “red-green-blue color sensors” in its two “eyes”, along with an embedded control system. Upon detecting a specific color, the sensor communicates with the computer system, which subsequently applies voltage to the scales. The magnitude and duration of the voltage are predetermined based on prior experimental outcomes and stored within the system program, thereby enabling autonomous color adjustments (Figure 6e). However, it is noteworthy that due to limitations in the color sensing components, the current system is constrained to transitioning solely between red, green, and blue colors, impeding its broader applicability in adaptive camouflage.

### 3.3. Film Interference Modulation

**Figure 7:**
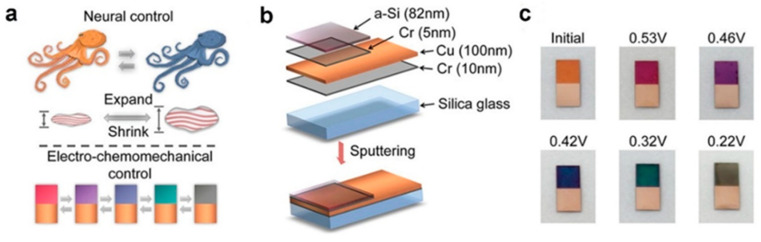
(**a**) The reversible coloration processes. (**b**) Schematic of the layered structure of the controllable coloration film. (**c**) Photographs of controllable coloration films at different voltages during the Li intercalation process. (**a**–**c**) Reproduced with permission [90]. Copyright (2018) Wiley-VCH.

Cephalopods predominantly manifest structural colors based on the principle of film interference [59]. Drawing inspiration from this natural phenomenon, Bao et al. innovatively engineered an electrochemically controllable interference color film by depositing a nanoscale layer of amorphous silicon (a-Si) on a reflective metal substrate (Figure 7a) [90]. The structural composition of the film, as depicted in Figure 7b, incorporates a chromium (Cr) layer primarily to enhance adhesion between the glass substrate, copper layer, and a-Si layer. Notably, the thin Cr layer exerts negligible influence on the optical properties of the entire film. Film interference ensues due to the reflection of light rays from the a-Si layer and the interface between the a-Si layer and the copper layer. Silicon, serving as electrode material in lithium-ion batteries, undergoes substantial volume changes throughout the lithiation/delithiation cycling process, concomitant with alterations in its refractive index. Consequently, under voltage control, the chemical composition and film thickness undergo concurrent changes during the electrochemical process, thereby resulting in the dynamic display of various colors (Figure 7c).

## 4. The Application of Environmental Fusion Modulation

The concept of “Environmental Fusion Modulation” refers to a technique used in the development of camouflage or adaptive materials, particularly in the context of mimicking natural environments. It likely involves the modulation or adjustment of certain properties of the material, such as color, texture, or transparency, to blend seamlessly with different environmental conditions or backgrounds. The term “fusion” suggests the merging or integration of these properties to achieve effective camouflage. Researchers have demonstrated significant progress in engineering bioinspired structural color materials, enabling dynamic color changes by manipulating the structural parameters of photonic crystals, plasmonic resonances, or film interference conditions. Despite these advancements, a notable deficit persists in seamlessly integrating and adapting these materials to the surrounding environment. Environmental responsiveness, encompassing factors such as changes in light intensity, temperature, and humidity, is essential for achieving optimal camouflage effectiveness across diverse natural settings. Addressing this gap requires innovative approaches that enable biomimetic materials to autonomously respond to environmental cues, ensuring adaptive coloration that seamlessly blends with the surroundings. Such advancements hold great promise not only for military applications but also for diverse fields such as architecture, design, and consumer electronics, where dynamic color-changing materials can enhance functionality and aesthetics in response to environmental stimuli.

**Figure 8:**
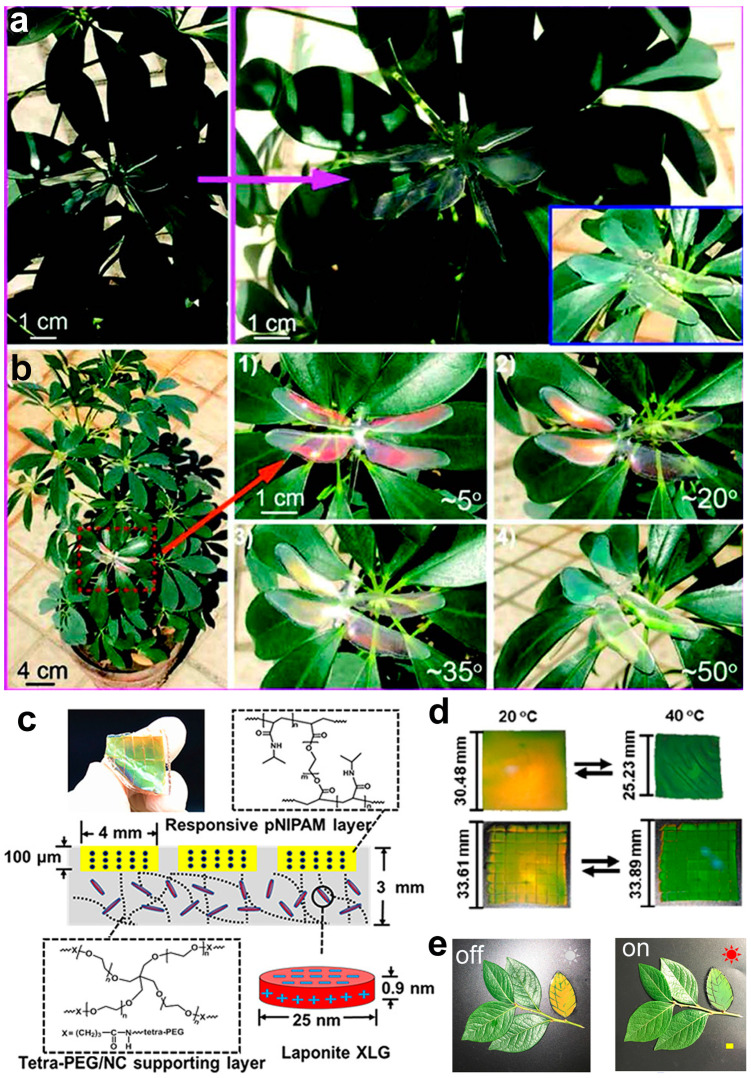
(**a**) Digital images of the biomimetic dragonfly film in the shadow of leaves. (**b**) Digital images of the biomimetic dragonfly film on the leaves. Taking at different angles in the sunlight. (**a**,**b**) Reproduced with permission. Copyright (2019) The Royal Society of Chemistry. (**c**) Photograph and schematic of SASS that includes dimensions and chemical structure of polymers. (**d**) Digital images of conventional responsive PNIPAM PC film and SASS in the same state. (**e**) Camouflaged “leaf”. (**c**–**e**) Reproduced with permission [93]. Copyright (2019) American Chemical Society.

Taking inspiration from the transparent wings of insects, which exhibit vibrant structural colors on the black background but retain their pigmentation pattern on the white background [91], Meng et al. utilized polymethyl methacrylate (PMMA, *n* = 1.49) as the assembly unit to construct ordered opal photonic crystal structures. These structures were subsequently infused with polydimethylsiloxane (PDMS, *n* = 1.41) to craft a biomimetic dragonfly film [92]. The small refractive index difference (Δ*n* = 0.08) renders this transparent biomimetic membrane nearly imperceptible in shaded areas caused by leaf obstruction (Figure 8a). In direct sunlight, the absence of bright structural colors from non-mirror angles is observed. Notably, the manifestation of vibrant structural colors is contingent upon the alignment of incident light and observation angles, displaying significant angular dependence (Figure 8b).

Salaita’s research team devised a strain-accommodating smart skin (SASS) employing responsive photonic crystal films and a tetra-polyethylene glycol support layer [93]. The responsive photonic film, synthesized via UV-curing of a precursor solution containing magnetic particles Fe_3_O_4_@SiO_2_ with a core–shell structure and poly(N-isopropylacrylamide) (PNIPAM), is integrated with a support layer formed through room temperature gelation of a precursor solution containing two tetra-polyethylene glycol prepolymers, tetra-PEG-NH_2_ and tetra-PEG-NHS. Demonstrating remarkable tensile strength and stretchability (Figure 8c). This system diverges from conventional temperature-responsive films by showcasing strain adaptability (Figure 8d). To illustrate its efficacy, a leaf-shaped SASS sample was arranged alongside real leaves, as illustrated in Figure 8e. Following exposure to sunlight, the color of the SASS “leaf” transitioned from orange to green, blending seamlessly with the real leaves. However, it is noteworthy that this system exhibits limitations in terms of sensitivity, and the color change does not occur instantaneously in real-time. Shang et al. devised a magnetic field-responsive fiber by embedding ethylene glycol droplets containing Fe_3_O_4_@C magnetic nanoparticles into PDMS [94]. In the presence of an external magnetic field, the dispersed Fe_3_O_4_@C nanoparticles rapidly assemble into one-dimensional chain-like structures, transforming the fiber color from brown to yellow-green, effectively matching the background color of green leaves and achieving adept camouflage. Upon removing the magnetic field, the yellow-green color dissipates, revealing the original color.

**Figure 9:**
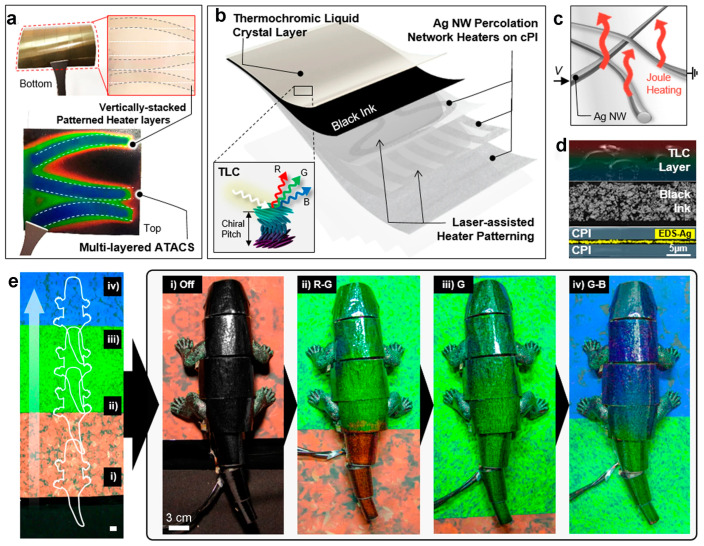
(**a**) Digital photos of multiple layers of ATACS and vertically stacked patterned heating layers. (**b**) Schematic diagram of the ATACS structure. (**c**) Schematic diagram of the joule heating mechanism. (**d**) The cross-sectional SEM of the ATACS. (**e**) The camouflage device under the moving chameleon model. Scale bar: 3 cm. Reproduced with permission [95]. Copyright (2021) The Author(s).

Despite the efficiency of this dynamic change controlled by a magnetic field, its practical application for adaptive camouflage is constrained. Recognizing these limitations, Kim et al. integrated thermochromic liquid crystal (TLC) ink with vertically stacked multilayer silver nanowire heaters to develop a pragmatic, scalable, and high-performance artificial chameleon skin (ATACS) (Figure 9a) [95]. The skin’s composition is delineated in Figure 9b. The color transition in the thermochromic liquid crystal is orchestrated through Joule heating facilitated by the multilayer silver nanowire heaters (Figure 9c,d). Augmented by an active control system and sensing units, this skin, when applied to a chameleon robot, can promptly adjust its color according to the background color (Figure 9e), positioning it as a promising candidate for adaptive camouflage devices.

## 5. Conclusions and Prospects

This review provides the fundamental mechanisms governing structural colors observed in natural organisms. Our primary emphasis centers on elucidating the bioinspired materials with tunable structural colors, alongside recent advancements in applications involving environmental fusion modulation.

Despite notable advancements, several challenges persist in the realm of bioinspired structural color in camouflage. These challenges encompass environmental stability, necessitating the assurance of long-term durability and stability of bioinspired structures under battlefield conditions, including exposure to light, moisture, and mechanical stress. Additionally, the integration and compatibility of bioinspired structures into existing materials, devices, and manufacturing processes require meticulous attention to compatibility considerations, performance trade-offs, and design constraints. Moreover, achieving scalability and cost-effectiveness in the large-scale fabrication of bioinspired structures with precision and efficiency remains a formidable hurdle, particularly for applications demanding mass production.

Future research avenues in bioinspired structural color are poised to concentrate on several key domains. Firstly, there is a need to shift focus towards bioinspired design, where the current emphasis on manipulating color hue (wavelength) should be expanded to encompass brightness (reflectance) and saturation (half-width at half maximum), thus facilitating comprehensive advancements in camouflage technology. Secondly, the development of wireless adaptive, intelligent structural color systems for camouflage warrants further investigation. This entails the integration of electrically, thermally, or optically responsive materials with periodic nanostructures to modulate color changes, alongside the employment of multi-layered architectures to fabricate actuators. Micro-electro-mechanical Systems (MEMS) offer precise control over the structural elements driving these color transformations. Furthermore, the incorporation of various sensors—including those for light, temperature, humidity, and proximity—coupled with wireless communication modules (e.g., Bluetooth, Wi-Fi), facilitates real-time monitoring and dynamic adjustment of the camouflage system. Thirdly, efforts should be directed towards enhancing multifunctionality, and expanding the utility of bioinspired structures beyond color control to include functionalities such as sensing, actuation, and energy harvesting. Lastly, interdisciplinary collaboration is imperative, fostering synergy among researchers from diverse disciplines such as materials science, optics, biology, and engineering to address multifaceted challenges and propel innovation in the field.

In summary, bioinspired structural color holds significant promise for the development of cutting-edge materials, devices, and systems with unparalleled color manipulation capabilities. Harnessing the synergistic interplay between structural and pigment colors is paramount in the quest for materials characterized by high controllability, weather resilience, and sensitivity. Moreover, the integration of autonomous control systems with color sensing units emerges as a critical aspect in the fabrication of adaptive camouflage devices capable of dynamically adjusting color across the entire spectrum.

## Data Availability

Data will be made available on request.
